# Host-Microbe Metagenomics: a Lens To Refocus Our Perspective on Infectious and Inflammatory Diseases

**DOI:** 10.1128/mSystems.00404-21

**Published:** 2021-08-17

**Authors:** Katrina L. Kalantar, Charles R. Langelier

**Affiliations:** a Chan Zuckerberg Initiative, San Francisco, California, USA; b Division of Infectious Diseases, University of California San Francisco, San Francisco, California, USA; c Chan Zuckerberg Biohub, San Francisco, California, USA

## Abstract

A dynamic relationship involving pathogen, host immune response, and microbiome characterizes the biological framework of many infectious and inflammatory diseases. Combined host/microbe metagenomics (mNGS) enables simultaneous assessment of all three features, enabling the study and diagnosis of diverse infectious and inflammatory processes ranging from pneumonia to sepsis to inflammatory diseases such as rheumatoid arthritis. Host/microbe mNGS holds promise for new mechanistic insights, diagnostic approaches, and precision medicine interventions.

## COMMENTARY

Infections represent a dynamic relationship between pathogen, host immune response, and local microbiome. Despite this, each aspect has historically been evaluated individually—through studies focused on clinical microbiology (1), immunology (2), or the bacterial microbiome (3)—leaving gaps in understanding of pathogenesis mechanisms and providing a narrow perspective on the systems biology of a given disease. Combined host/microbe metagenomics (mNGS) addresses this gap by enabling broad profiling of all three key components of infectious diseases. Its utility spans across diverse disease profiles, ranging from lower respiratory tract infections (LRTI) to sepsis to gastrointestinal disease to meningitis, where the goal of clinical diagnostics is ultimately to determine which patients have an infection and what is causing the infection. This holds potential to inform precision treatment that can reduce the need for broad-spectrum antibiotics.

## BACKGROUND ON mNGS

mNGS enables culture-independent, unbiased profiling of microbial and host-derived nucleic acid directly from biological samples. DNA-based mNGS has been used for over a decade to detect bacterial pathogens and study the human microbiome (3, 4). Transcriptome sequencing (RNA-seq) expands this utility by also enabling detection of RNA viruses (5) while still permitting detection of other pathogens (6).

As opposed to stool specimens used to study the gut microbiome, nucleic acid obtained from other anatomical sites, such as the respiratory tract, is derived mostly from host and not from microbial origin, even in the setting of active infection (5). Thus, both DNA sequencing (DNA-seq) and RNA-seq can be used to identify heritable single nucleotide polymorphisms (SNPs) associated with disease (7). A unique attribute of RNA-seq, however, is the capacity to detect variations in host gene expression levels, thereby enabling combined host and microbial profiling (5).

## APPLICATION OF HOST/MICROBE mNGS TO COVID-19 AND OTHER LRTI

The challenges associated with determining the cause of LRTI are particularly well suited for the application of mNGS. Even before the coronavirus disease 2019 (COVID-19) pandemic, LRTI were the leading cause of death from infectious disease globally, and a top cause of death by any cause in low and middle income countries (8). Difficulties identifying, and thus appropriately treating, the etiologic microbes contributes significantly to the burden of LRTI, as the responsible pathogens frequently remain undetected due to the limitations of existing clinical diagnostics, which rely heavily on bacterial culture. Further compounding this issue are challenges in accurately distinguishing LRTI from other inflammatory lung diseases, as many conditions mimic infection, especially in critically ill patients with systemic illness.

In the first proof-of-concept demonstration of host/microbe mNGS for diagnosis of LRTI (5), we identified host transcriptional signatures and features of the lung microbiome that enabled accurate identification of infection and differentiation from noninfectious acute respiratory diseases. In a complex population of critically ill patients requiring mechanical ventilation, traditional clinical metrics such as fever and white blood cell count were unable to distinguish LRTI from other causes of respiratory failure at the time of hospital admission, while in contrast, host and microbial mNGS classifiers could do so with high accuracy (2).

This improved accuracy for identification of infection is driven by the fact that host and microbial data provide complementary information that can assist with contextualization that is critical to the ability to disambiguate cases of infection. Specifically, it is possible to interpret the set of microbes identified in a particular case within the context of host biology and vice versa to illuminate interactions that may be indicative of infection. Incidental carriage of potentially pathogenic microbes in the respiratory tract is common, and thus differentiating pathogens from commensals is a major challenge for existing microbiologic diagnostics. Profiling the host response provides context to microbes identified, informing the presence of infection versus just incidental pathogen colonization (5).

Complementing this host-based pathogen contextualization is the ability to assess lung microbiome composition, which becomes disrupted during LRTI, with loss of diversity and pathogen dominance correlating with infection (5, 9). Using a machine learning model trained on metagenomic data from patients with either proven LRTI or noninfectious respiratory illnesses, we found that mNGS could accurately identify pathogens—including viruses, bacteria, and fungi—amid a background of more abundant predominantly bacterial commensals. The importance of this contextualization is paramount given the increasing use of highly sensitive molecular assays for clinical infectious disease diagnosis.

Emergence of severe acute respiratory syndrome coronavirus 2 (SARS-CoV-2) has highlighted the value of an unbiased diagnostic assay capable of detecting novel, emerging pathogens, as indeed it was mNGS that illuminated the etiology of an idiopathic pneumonia syndrome in Wuhan, China, in December 2019 (10). Further work has leveraged mNGS to not only identify coinfecting pathogens and track the emergence of new variants but has also illuminated unique aspects of the host immunologic response to SARS-CoV-2 that differ markedly from other viral pathogens (11, 12).

For instance, recent work by our group and others used host/microbe mNGS to demonstrate that early SARS-CoV-2 infection is characterized by attenuated innate immune signaling in the upper respiratory tract (11). One of the defining features of COVID-19 is peak viral load—and infectiousness—at or prior to the onset of symptoms, a feature that has made control of coronavirus transmission extremely difficult and likely contributed to the emergence of a pandemic far beyond the scale of what was experienced with the original SARS virus in 2003. Suppressed immune signaling provides a potential explanation for how peak viral load could exist in the absence of symptoms.

## APPLICATION OF HOST/MICROBE mNGS TO OTHER INFECTIOUS AND INFLAMMATORY DISEASES

The potential of host/microbe mNGS to advance understanding of respiratory disease biology extends far beyond LRTI. Compelling work demonstrates that both dysregulated host response and disrupted microbial components characterize the acute respiratory distress syndrome (ARDS) (3), chronic obstructive pulmonary disease, asthma, and interstitial lung disease. To date, immunologic and microbial aspects of these conditions have largely been examined independently. Host/microbe mNGS provides an opportunity to more holistically study and diagnose these conditions and to understand clinical subphenotypes (13) with unique clinical features or treatment responses.

While the respiratory tract is particularly well suited for host-microbe mNGS-based assessment, the utility of this approach without doubt extends to other systems and disease states. Sepsis, for instance, is a disease defined as a dysregulated host response to infection (14). Despite this, nearly all available sepsis diagnostics are designed to detect microbes, although recent advancements have led to the development of accurate host-based transcriptomic classifiers (15). No diagnostic yet, however, has integrated unbiased pathogen detection with host response profiling for sepsis diagnosis.

Meningitis is well studied in the context of mNGS pathogen diagnostics (16, 17), which have proven exceptional in terms of detecting rare and uncommon microbial explanations for persistent central nervous system disease. Infectious etiologies of meningitis and encephalitis, however, can often be clinically undifferentiable from autoimmune neurologic disease, despite increasing knowledge of differing cell types (and therefore host gene expression patterns) across these etiologies. A clear opportunity exists for host-microbe mNGS to advance our ability to diagnose neuroinflammatory disease.

Recent work has demonstrated that the human gut microbiome may play a role in modulating the onset and perpetuation of pathological inflammatory signaling in chronic autoimmune diseases, such as rheumatoid arthritis (18, 19). Combining assessment of systemic and local host immune responses with unbiased profiling of microbial taxa, including bacteria, viruses, and fungi, holds promise for characterizing this relationship at a higher resolution. Without question, opportunities exist to leverage host/microbe mNGS to further understand the systems biology of autoimmune diseases, identify unique patient subphenotypes, and predict clinical outcomes.

## DEMOCRATIZATION OF HOST/MICROBE mNGS COMPUTATIONAL PIPELINES

Despite the potential for host/microbe mNGS to advance our understanding of infectious and inflammatory diseases, use has been limited by both the cost of sequencing and the computational infrastructure needed for computationally intensive bioinformatics analysis. Advancements in cloud-based open source pipelines such as the IDseq pipeline (20) have democratized access to, and analysis of, mNGS data. Now anyone in the world with Internet connectivity can access the expanding repositories of publicly accessible host and microbial sequencing data to study their disease of interest. As the cost of sequencing continues to decline precipitously, the utility of culture-independent host/microbe mNGS will continue to grow in resource-limited areas, potentially enabling a leap beyond traditional microbiology laboratories and their requisite infrastructure of incubators, culture media, sterilization instruments, and microscopes.

## FUTURE OUTLOOK

The COVID-19 pandemic has brought infectious disease systems biology to the forefront and has highlighted key roles for microbial metagenomics and host transcriptional profiling in both epidemiologic surveillance and in the study of emerging infections. When the pandemic subsides, we will have a renewed opportunity to refocus our traditional perspectives on other major infectious and inflammatory diseases—from pneumonia to sepsis to rheumatoid arthritis—through the lens of host/microbe metagenomics. This approach holds promise for new mechanistic insights, diagnostic approaches, and precision medicine interventions.
